# Sedentary lifestyle is independently associated with higher fat mass index regardless of physical activity level in patients with coronary artery disease

**DOI:** 10.1186/s12872-025-05368-2

**Published:** 2025-11-26

**Authors:** Marko Novaković, Diogo Salgueiro, Madalena Lemos Pires, Mariana Borges, Gonçalo Sá, Zlatko Fras, Fausto J. Pinto, Ana Abreu, Rita Pinto

**Affiliations:** 1 Department of Vascular Diseases, University Medical Centre Ljubljana, Zaloška cesta 7/VI, Ljubljana, 1000 Slovenia; 2 Faculty of Medicine, University of Ljubljana, Vrazov trg 2, Ljubljana, 1000 Slovenia; 3 Instituto de Medicina Preventiva e Saúde Pública, Faculdade de Medicina, Universidade de Lisboa, Avenida Professor Egas Moniz, Lisboa, 1649-028 Portugal; 4 Centro Cardiovascular da Universidade de Lisboa (CCUL@RISE), Centro Académico de Medicina de Lisboa, Faculdade de Medicina, Universidade de Lisboa, Avenida Professor Egas Moniz, Lisboa, 1649-028 Portugal; 5 Serviço de Cardiologia, Unidade Local de Saúde Santa Maria (ULSSM), Av. Prof. Egas Moniz MB, Lisboa, 1649-028 Portugal; 6 Instituto de Saúde Ambiental, Faculdade de Medicina, Universidade de Lisboa, Avenida Professor Egas Moniz, Lisboa, 1649-028 Portugal

**Keywords:** Cardiac rehabilitation, Obesity, Sedentary behavior, Physical activity, Coronary artery disease

## Abstract

**Background:**

Cardiac rehabilitation (CR) after a coronary artery disease (CAD) event is recommended to improve risk factor control, as some of them remain poorly controlled (e.g. obesity, physical activity), while new ones are emerging (e.g. sedentary behavior). The aim of our study was to investigate the associations between physical activity, sedentary behavior, and traditional risk factors with body fat mass index (FMI) in patients with CAD.

**Methods:**

In this cross-sectional study, CAD patients entering phase III CR (2016–2023) were assessed for FMI by dual-energy X-ray absorptiometry, cardiorespiratory fitness by cardiopulmonary exercise testing, 7-day accelerometer-measured physical activity and sedentary behavior, and cardiovascular risk factors.

**Results:**

There were 111 CAD patients, their average age was 61.7 ± 9.8 years, 15 (13.5%) were female. Patients were divided into three groups, based on the FMI: normal (*n* = 19), overweight (*n* = 62) and obese (*n* = 30). Obese patients, compared to patients with normal FMI, had significantly higher systolic blood pressure, total sedentary time, non-HDL and triglycerides levels, and lower exercise capacity. On the contrary, there were no significant differences in physical activity level, diastolic blood pressure, total cholesterol, HDL and LDL cholesterol. In a multivariate linear regression analysis, sedentary time showed an independent association with FMI, whereas physical activity did not.

**Conclusion:**

Obese CAD patients exhibit poorer control of blood pressure, lipid levels, and sedentary behavior compared with normal-weight and overweight patients. Effective strategies are needed to curb sedentary behavior in this population, which may directly reduce body fat.

## Introduction

Coronary artery disease (CAD) is the most prevalent form of cardiovascular disease (CVD) and is increasing in the middle-aged and elderly populations, being responsible in 2019 for the death of more than 18.0 million people worldwide [1]. Over the years, it has remained one of the leading causes of mortality and disability worldwide and its prevalence is expected to increase even more in the coming years [1–3]. Cardiac rehabilitation (CR) is a multidisciplinary intervention with several components designed to reduce cardiovascular (CV) risk, encourage healthy behaviors, mitigate physical impairment, and promote an active lifestyle. It is an essential part of contemporary heart disease care and a priority in countries with high prevalence of CAD [4–7]. Most CR programs are exercise-based and generally consist of three phases and act at the level of CV risk factors control, particularly those that are modifiable [7].

According to literature [5], phase II CR programs usually last about 3 months and are often the final step in structured rehabilitation. Phase III CR, in contrast, is community-based and intended for life-long secondary prevention and lifestyle support, yet only a minority of patients continue with it. This low participation may contribute to the persistence of poorly controlled risk factors, as shown in the EUROASPIRE surveys, where 82% of CAD patients remained overweight or obese 6–24 months after a CV event [8].

This highlights the need for a more precise assessment of adiposity, as body mass index (BMI) alone cannot differentiate between fat and lean mass. Fat mass index (FMI), derived from dual-energy X-ray absorptiometry (DXA), may therefore provide a more accurate characterization of obesity-related risk in this population. At the same time, sedentary behavior has emerged as an important but underexplored cardiovascular risk factor, at least equally important as physical activity, and appears to act independently, being associated with cardiovascular mortality [9, 10]. However, data on how movement behaviors (physical activity and sedentary time), traditional cardiovascular risk factors, and adiposity interact in patients after CR remain scarce.

The aim of our study was therefore to investigate the associations between traditional CV risk factors, physical activity, sedentary behavior, and FMI in patients with CAD entering a community-based phase III CR program.

## Methods

### Study design and clinical data

This cross-sectional study included patients enrolled in a Phase III CR program. All patients entered the phase III CR program of the Cardiac Rehabilitation Centre of Centro Hospitalar Universitário Lisboa Norte/Faculdade de Medicina da Universidade de Lisboa/CRECUL, located at the Estádio Universitário de Lisboa, between the year of 2016 and 2023.

Inclusion criteria were as follows: patients aged over 18 years, male and female, who had confirmed CAD based on angiography in at least one major epicardial vessel, clinical evidence of CAD, which can be in the form of previous myocardial infarction or coronary revascularization (coronary artery bypass graft or percutaneous coronary intervention), or angina pectoris.

Exclusion criteria included: lack of clinical information related to CVD, missing cardiopulmonary exercise testing information, insufficient data on body composition information or physical activity information.

Risk factors such as hypertension, dyslipidemia, diabetes mellitus, cigarette smoking, and conditions such as heart failure were assessed using the initial assessment questionnaire and verified through the patients’ clinical records, and their treatment was provided in accordance with the applicable guidelines at the time of inclusion.

The SMART (Second Manifestations of Arterial Disease) score was calculated for each patient to estimate their 10-year risk of recurrent cardiovascular events. The score incorporates traditional risk factors such as age, sex, blood pressure, lipid profile, diabetes status, smoking, and prior cardiovascular history [11]. A fasting blood sample (12–14 h of fasting) was collected to measure fasting blood sugar, serum lipids including triglycerides, and total-cholesterol using enzymatic colorimetric methods. HDL cholesterol was determined after dextransulphatemagnesiumchloride precipitation of non-HDL cholesterol; then, LDL cholesterol was calculated according to Friedewald formula [12].

### Clinical trial number

 Not applicable.

### Physical activity and sedentary behavior

Physical activity and sedentary behavior were assessed objectively by accelerometry (ActiGraph, wGT3X + BT, Florida, USA). Each patient was instructed to wear the ActiGraph wGT3X + BT accelerometer, which was attached to an elastic waist belt. The accelerometer was positioned in line with the axillary line of the right iliac crest and worn continuously for a period of seven days. The patient had to remove the accelerometer in water-based activities and during sleep. For a day to be considered valid, at least 600 min (10 h) of monitored wear time had to be measured. Patients who had a minimum of three valid days, including at least one weekend day, were included in the analysis. The accelerometers were set to raw mode with a frequency of 100 Hz and the data was later downloaded into 10-second epochs using the Actilife software version 6.9.1. To determine the time spent in different intensity periods and define valid recordings, the cut-off points and wear time validation criteria established by Troiano and colleagues were used [13]. These criteria were used as a reference to analyze and interpret the data collected from the accelerometers. Moderate to vigorous physical activity (MVPA) time, light intensity physical activity (LIPA) time, and sedentary time were obtained for each patient.

The classification of patients as physically active was done according to the most recent European Society of Cardiology guidelines [7].

### Body composition

All patients were tested in the morning following a 12-hour fast and refrained from caffeine, alcohol, and MVPA during the last 24-h and all the following anthropometric procedures were led by the same certified technician. Height and weight were measured using an electronic scale with a stadiometer (SECA, Hamburg, Germany) and body mass index (BMI) was calculated (kg/m^2^). Whole and regional body mass (bone mineral content, lean soft tissue, and fat mass) was estimated using DXA (Hologic Explorer-W, fan-beam densitometer, software QDR for Windows version 12.4, Hologic, USA). Due to its independence of lean mass, we have decided to use the fat mass index as a measure of the fat quantity in the body. It is derived by dividing total fat mass by height squared.

Fat mass index (FMI) characterization was classified according to the following criteria [14]:

- In men: FMI > 6.0 kg/m^2^ indicates overweight and FMI > 9.0 kg/m^2^ indicates obesity.
- In women: FMI > 9.0 kg/m^2^ indicates overweight and FMI > 13.0 kg/m^2^ indicates obesity.

### Cardiopulmonary Exercise Testing (CPET)

CPET was performed to assess aerobic capacity and overall cardiorespiratory fitness. A symptom-limited, ramp incremental CPET was performed on a cycle ergometer. Peak oxygen uptake (VO₂peak) was defined as the highest 30-s value achieved during the last minute of exercise. Continuous ECG and repeated blood pressure measurements were recorded, and the VE/VCO₂ slope and oxygen were determined [15].

### Statistical analysis

Normality and homogeneity were tested using Kolmogorov-Smirnov test and Levene test, respectively. Variables were expressed as means ± standard deviation for normally distributed variables, while asymmetrically distributed variables were expressed as median (quartile 25 – quartile 75). Asymmetrically distributed variables were log-transformed before further analysis, where appropriate. For the comparison analysis of baseline qualitative variables, the independent samples T-Test and ANOVA with Tukey post-hoc test were used to compare 2 and 3 groups, respectively, without adjustment for potential confounders such as age, sex, medications, or other cardiovascular risk factors. Categorical variables were estimated as frequencies and compared using the Chi-square Test. For indicating predictors, multivariate linear regression analysis was used. Statistical significance was set at a level of 0.05. All data was analyzed using Statistical Package for the Social Sciences (SPSS) version 26.0 software (IBM SPSS Statistics, Chicago, IL, USA).

## Results

There were 111 CAD patients included in our study, their average age was 61.7 ± 9.8 years, 15 (13.5%) were female, median FMI was 7.8 kg/m^2^ (Q1 6.7 – Q3 9.5). Patients were divided into three groups, based on the FMI characterization, as follows: normal, overweight, and obesity (Table 1). There were no significant differences among groups in terms of age, diastolic blood pressure, height, physical activity levels, and other risk factors for CVD (Tables 1 and 2). However, there was a significant difference among groups in terms of sex distribution (*p* = 0.041).

**Table 1:** Baseline demographics and clinical data

	All (*n* = 111)	Normal FMI (*n* = 19)	Overweight (*n* = 62)	Obese (*n* = 30)	*p*
Age, years	61.7 (9.8)	58.9 (10.6)	61.9 (10.3)	63.0 (7.9)	0.349
Females, n (%)	15 (13.5)	5 (26.3)	4 (6.5)	6 (20.0)	0.041
Height, cm	169 (9)	169 (9)	169 (8)	169 (10)	0.967
Weight, kg	78.4 (13.2)	66.4 (10.3)^*#^	76.1 (8.9)^#◊^	90.5 (13.2)^*◊^	< 0.001
BMI, kg/m^2^	27.4 (3.7)	23.1 (1.8)^*#^	26.7 (2.0)^#◊^	31.7 (3.1)^*◊^	< 0.001
FMI, %	7.8 (6.7–9.5)	5.7 (4.6–5.9)^*#^	7.5 (6.9–8.3)^#◊^	11.1 (9.7–13.1)^*◊^	< 0.001
VO2peak, L/min	1.71 (1.30–1.97)	1.71 (1.23–2.11)	1.75 (1.31–2.05)	1.61 (1.33–1.85)	0.516
VO2peak, ml/kg/min	21.9 (6.6)	25.2 (7.7)^*^	22.9 (6.3)^◊^	17.7 (4.3)^*◊^	< 0.001
% of predicted VO2peak, %	80.3 (20.6)	89.3 (22.4)^*^	83.5 (18.2)^◊^	68.1 (19.3)^*◊^	< 0.001
Workload, W	147.8 (48.3)	155.6 (46.5)	151.5 (49.8)	135.2 (45.2)	0.236
RER	1.17 (0.09)	1.18 (0.12)	1.17 (0.08)	1.16 (0.09)	0.159
Oxygen pulse, mL/beat	13.2 (3.3)	11.9 (3.0)	13.5 (3.3)	13.3 (3.2)	0.700
% of predicted Oxygen pulse, %	98.6 (22.1)	102.8 (24.9)^*^	102.4 (19.7)^◊^	88.0 (22.4)^*◊^	0.008
VE/VCO2	32.2 (8.2)	30.6 (8.7)	32.5 (8.5)	32.3 (7.6)	0.620
HRpeak, bpm	130 (21)	141 (21)^*^	130 (20)	123 (20)^*^	0.011
Systolic blood pressure, mmHg	113 (106–122)	104 (94–120)^*^	111 (106–120)^◊^	120 (112–130)^*◊^	0.009
Diastolic blood pressure, mmHg	7 − 0 (60–76)	70 (60–80)	70 (60–74)	73 (60–78)	0.581
SMART risk score^҂^, %	11.8 (8.3–17.4)	10.3 (6.4–11.8)^*^	12.6 (8.6–18.0)	13.4 (8.2–18.4)^*^	0.038
Total cholesterol^҂^, mg/dL	138 (119–153)	129 (120–143)	140 (113–152)	145 (126–166)	0.129
HDL cholesterol^҂^, mg/dL	45 (37–51)	49 (44–59)	44 (36–49)	45 (39–53)	0.069
LDL cholesterol^҂^, mg/dL	67 (53–86)	61 (51–73)	66 (54–86)	72 (56–94)	0.194
Non-HDL cholesterol^҂^, mg/dL	91 (72–109)	78 (63–96)^*^	88 (73–108)	97 (80–129)^*^	0.032
Triglycerides^҂^, mg/dL	100 (73–129)	91 (69–110)^*^	100 (73–134)	100 (79–160)^*^	0.042
Diabetes mellitus	21 (18.9%)	1 (5.3%)	13 (21.0%)	7 (23.3%)	0.239
Cigarette smoking					0.137
- never	29 (26.1%)	5 (26.3%)	17 (27.4%)	7 (23.3%)	
- former	71 (64.0%)	14 (73.7%)	35 (56.5%)	22 (73.3%)	
- current	11 (9.9%)	0 (0%)	10 (16.1%)	1 (3.3%)	
Family history of CVD	54 (48.6%)	11 (57.9%)	28 (45.2%)	15 (50.0%)	0.614
Completed phase II CR	97 (87.4%)	17 (89.5%)	54 (87.1%)	26 (86.7)	0.954
Myocardial infarction	85 (76.6%)	15 (78.9%)	47 (75.8%)	23 (76.7%)	0.961
Left ventricular ejection fraction, %	54.4 (10.4)	57.2 (12.7)	54.5 (9.6)	52.6 (10.5)	0.330
Heart failure	12 (10.8%)	1 (5.3%)	5 (8.1%)	6 (20%)	0.156
ACEi/ARB (%)	65 (59%)	10 (53%)	34 (55%)	21 (70%)	0.325
Beta blockers, n (%)	88 (79%)	12 (63%)	50 (81%)	26 (87%)	0.130
SGLT2 inhibitors, n (%)	29 (26%)	4 (21%)	16 (26%)	9 (30%)	0.783
MRA, n (%)	11 (10%)	1 (5%)	6 (10%)	4 (13%)	0.651
Statins, n (%)	104 (94%)	17 (90%)	59 (95%)	28 (93%)	0.669

*ACEi/ARB * Angiotensin-converting enzyme inhibitors and angiotensin receptor blockers, *BMI* Body mass index, *CR* Cardiac rehabilitation, *CVD* Cardiovascular disease, *FMI* Fat mass index, *SGLT2* Sodium-glucose cotransporter 2, *MRA* Mineralcorticoid receptor antagonists, *RER* Respiratory exchange ration, *SMART* Second manifestation of arterial disease, *VE/VCO2* Minute ventilation/carbon dioxide production slope, *VO*_*2*_ *peak * peak oxygen consumption

^*^p < 0.05 for normal vs. obese

^҂^- *n* = 106

^#^*p* < 0.05 for normal vs. overweight

^◊^*p* < 0.05 for overweight vs. obese

**Table 2:** Accelerometer metrics across fat mass index groups

	All (*n* = 111)	Normal FMI (*n* = 19)	Overweight (*n* = 62)	Obese (*n* = 30)	*P*
Total wearable time, min/day	875 (826–925)	842 (808–902)	876 (827–926)	881 (833–942)	0.181
MVPA, min/week	294 (206–462)	348 (217–550)	296 (228–456)	249 (182–420)	0.197
Average MVPA, min/day	44 (29–66)	51 (31–79)	45 (33–65)	36 (27–57)	0.118
LIPA, min/week	1023 (863–1309)	964 (733–1226)	1026 (867–1322)	1038 (864–1355)	0.375
Average LIPA, min/day	148 (124–187)	145 (114–176)	148 (124–189)	148 (123–199)	0.721
Sedentary time per week, min/week	4606 (4193–5063)	4372 (4188–4612)^*^	4651 (4125–5119)	4773 (4315–5196)^*^	0.037
Sedentary time per day, hours/day	11 (10–12)	11 (10–11)	11 (10–12)	11 (10–12)	0.103
Sedentary time per day, minutes/day	663 (613–719)	656 (599–689)	664 (609–731)	680 (627–742)	0.139

*FMI *Fat mass index, *LIPA *Light intensity physical activity, *MVPA *Moderate-to-vigorous physical activity

^*^p < 0.05 for normal vs. obese

### Exercise capacity and SMART risk score

Obese patients had significantly lower exercise capacity compared to patients with normal FMI and overweight (17.7 ± 4.3 vs. 22.9 ± 4.3 vs. 25.2 ± 7.7 ml/kg/min, *p* < 0.001). Obese patients also had higher SMART risk scores compared to patients with normal FMI (13.4 [8.2–18.4] vs. 10.3 [6.4–11.8] %, *p* = 0.038).

### Blood pressure

Resting systolic blood pressure was higher in obese patients than in normal FMI patients (120 [112–130] vs. 104 [94–120] mmHg, *p* = 0.009). No significant differences were observed in diastolic blood pressure among the groups.

### Physical activity and sedentary behavior

Total sedentary time was greater in obese patients than in normal FMI patients (4773 [4315–5196] vs. 4372 [4188–4612] min/week, *p* = 0.037). Physical activity levels did not differ significantly among groups (Table 2).

### Lipid parameters

Significant differences were observed in lipid status among groups. Obese patients had higher non-HDL cholesterol compared to normal FMI patients (97 [80–129] vs. 78 [63–96] mg/dL, *p* = 0.032) and higher triglyceride levels (100 [79–160] vs. 91 [69–110] mg/dL, *p* = 0.042).

We have further performed multivariate linear regression analysis using FMI as a dependent variable (Table 3) and demographics, presence of heart failure, exercise capacity, physical activity level, and sedentary time as independent variables.

**Table 3:** Multivariate linear regression analysis using log fat mass index as a dependent variable

	Unstandardized Coefficients		Standardized Coefficients	T	Sig.	95% Confidence interval for B	
	B	Std. Error	Standardized Beta			Lower bound	Upper bound
(Constant)	−0.436	0.336		−1.298	0.197	−1.102	0.230
Age	0.001	0.001	0.103	1.306	0.195	−0.001	0.003
Sex (male)	0.200	0.025	0.527	7.829	< 0.001	0.149	0.250
Heart failure	0.024	0.025	0.058	0.974	0.332	−0.025	0.074
Weight	0.007	0.001	0.733	11.522	< 0.001	0.006	0.008
Log VO_2_ peak (ml/min)	−0.125	0.086	−0.126	−1.460	0.147	−0.295	0.045
Log Moderate to vigorous physical activity level (minutes/day)	−0.007	0.034	−0.013	−0.203	0.840	−0.075	0.061
Log Light physical activity level (minutes/day)	0.047	0.063	0.045	0.751	0.454	−0.078	0.172
Log Sedentary time (minutes/day)	0.211	0.098	0.127	2.154	0.034	0.017	0.406

Dependent Variable: Log Fat Mass Index

Model fit: Adjusted R^2^ = 0.638; *p* < 0.001

The regression analysis has shown that average sedentary was significantly associated with FMI, adjusted for age, sex, weight, and exercise capacity. Interestingly, neither MVPA nor LIPA levels were significantly associated with FMI (Table 3).

## Discussion

In the present study, we focused on poorly controlled CV risk factors and assessed them with objective methods – physical activity and sedentary behavior using accelerometers, and body composition using DXA. Obese patients had poorer controlled systolic blood pressure, lower exercise capacity, increased levels of sedentary behavior, and higher levels of triglycerides and non-HDL cholesterol. Furthermore, sedentary time was associated with higher values of body fat (expressed as FMI) irrespective of age, sex, body weight, heart failure, exercise capacity, LIPA, and MVPA level.

Our results have shown that 82.9% of our patients were overweight or obese, which is in line with results from the large multicentric EUROASPIRE V registry, in which 82% of patients after a CV event were overweight or obese [16], despite the greater percentage of attendance to the phase II CR program in our sample (87% compared to only 32% in the EUROASPIRE V registry), likely because our cohort included patients voluntarily entering CR and thus particularly motivated to engage in their health. Furthermore, the relatively high median LDL value (67 mg/dL), with no differences between groups, may reflect the shift in target thresholds over time (from < 70 mg/dL until 2019 to < 55 mg/dL thereafter [16]), as well as the well-documented difficulty of achieving adequate lipid control in CAD patients. This is consistent with findings from the EUROASPIRE V registry, where 71% of patients across Europe (and 69% in Portugal) did not reach LDL levels below 70 mg/dL [16].

Associations between obesity and other CV risk factors have been extensively researched. Higher systolic blood pressure in obese patients may be a consequence of the interplay of various mechanisms, including the renin-angiotensin-aldosterone system, central and sympathetic nervous system, renal dysfunction, adipokines, and gastrointestinal hormones [17]. Numerous studies have indicated obesity being a major risk factor for hypertension [18–20]. Surprisingly, most patients in our sample had well-controlled blood pressure, likely as a result of antihypertensive therapy. Nevertheless, although median blood pressure values were within the target range, obese patients exhibited slightly, yet significantly, higher systolic blood pressure, which is consistent with the mechanisms described above.

Also, in our sample obese and overweight patients had significantly higher risk for 10-year risk of myocardial infarction, stroke, or CV death compared to patients with normal FMI. Further studies are needed to examine how this combination of risk factors affects the incidence of new CV events in patients with already established CAD. Furthermore, different weight loss strategies are needed to answer whether the weight loss is associated with a reduction in clinically relevant events.

FMI provides important clinical information beyond traditional measures such as BMI [21]. This distinction is particularly relevant in patients with CAD, where excessive fat mass—rather than overall body weight—has been shown to contribute to adverse outcomes [22]. Incorporating FMI into clinical assessment therefore enables a more precise evaluation of body composition, helps to identify patients at risk despite having a normal BMI, and may guide individualized therapeutic strategies in rehabilitation medicine. In this way, FMI can contribute to tailoring exercise prescriptions, nutritional interventions, and follow-up care more effectively than widely used BMI.

Significant differences in some lipid profile parameters, e.g. non-HDL cholesterol and triglycerides, in obese vs. normal FMI individuals, were already shown in the literature reports [23]. Hypertriglyceridemia is the hallmark of dyslipidemia in obesity and may be an important cause of other lipid abnormalities [24]. Non-HDL cholesterol, on the other hand, accounts for all the atherogenic particles in the lipid metabolism, and its values are important predictors of CAD events regardless of triglyceride values [23]. These significant differences in blood pressure and some blood lipid levels between obese patients and patients with normal FMI were summed through the SMART risk score, calculated to predict 10-year combined major adverse events, defined as non-fatal myocardial infarction, non-fatal stroke, or vascular death in patients with already established CAD [11, 25]. According to our results, obese patients have significantly higher SMART risk scores compared to those with normal FMI.

At first glance, our results show that obese patients had markedly reduced peak exercise capacity and percentage of predicted exercise capacity. However, this apparent underestimation is likely due to traditional normalization by total body weight rather than fat-free mass, as previously discussed in the literature [26]. As VO₂peak normalized by fat-free mass was not available, this should be acknowledged as a limitation, since body weight normalization may overstate differences in exercise capacity between groups. The lack of significant differences in absolute (non-normalized) maximal oxygen uptake and workload supports this interpretation.

Another intriguing result is that sedentary time was significantly associated with FMI, whereas physical activity in both the moderate-to-vigorous and light intensity ranges was not, highlighting a new and important observation. Our results are only partially in line with the recently published ProPASS study on the general population, in which sedentary behavior, but also MVPA and LIPA, were associated with waist circumference and BMI [27]. In a study with CAD patients, LIPA was associated with decreased waist circumference and body mass index [28]. Although sedentary behavior emerged as a significant risk factor for subclinical and clinical CVD development [29–32] and mortality [33] in the general population, there is less data on its effects on the parameters of CV health in patients with established CAD [34]. Different CR programs after CV events did not succeed in decreasing sedentary time [35], even dedicated interventions aiming to reduce sedentary behavior did not induce greater reduction in sedentary time as compared to controls [36]. As much as 87% of our patients completed phase II CR, which probably improved their physical activity levels before entering phase III, as 91% reached the guidelines recommendation of MVPA levels [7]. Interestingly, no significant differences in MVPA were observed between groups; however, there was a non-significant trend toward slightly higher levels of LIPA in obese patients compared with others. This observation warrants further investigation. Nevertheless, the incidence of overweight and obese patients remained high, and regression analyses showed that more sedentary hours were associated with higher FMI. Potential physiological mechanisms linking sedentary behavior to higher FMI in patients with coronary artery disease include the suppression of lipoprotein lipase activity in skeletal muscles during prolonged sedentary periods, which favors fat accumulation [37]. Another potential mechanism underlying these findings could be myosteatosis, which has been described in patients with heart failure with preserved ejection fraction, particularly among those with obesity [38], and may similarly be present in obese patients with coronary artery disease. It is conceivable that periods of increased physical activity (e.g., during phase II CR) followed by a return to prolonged sedentary behavior may exacerbate fat deposition, as observed in studies on older patients with heart failure [39]. In addition, sedentary behavior may blunt glucose uptake in muscle tissue, promoting insulin resistance and further adiposity [40]. It seems that these, along with other less well-characterized mechanisms, may be particularly pronounced during the sedentary portion of the activity continuum, resulting in a stronger association between prolonged inactivity and FMI than with physical activity levels.

These findings suggest that rehabilitation and lifestyle modification strategies should aim not only to increase structured physical activity, which most CR programs achieve, but also to reduce overall sedentary behavior, as these are complementary targets rather than mutually exclusive. These actions should not be equivalent (as more MVPA is probably not associated with less sedentary hours) but complementary, which requires future studies. Specific strategies to reduce sedentary time could include active workstations at the workplace, “active sitting” with pedal devices in offices, and many others, together with digital reminders or mobile applications prompting patients to stand or move periodically throughout the day [41, 42]. Also, prospective studies are needed to answer the question of whether the sedentary time reduction would be associated with weight loss and reduction in body fat.

We have identified some limitations in our study. Firstly, the sample size. Although the number of included patients was comparable to other reports in this field, larger prospective multicenter studies are needed to confirm our findings; thus, this work should be regarded as a pilot study to be followed by research in a substantially larger cohort. Secondly, the percentage of female patients. Even though in most studies on patients with CAD, there are 20% or fewer females, our results must be carefully interpreted due to this underrepresentation. Thirdly, although accelerometers provide objective measurements, which do not always correlate with questionnaire-based assessments [43], some overestimation of physical activity and underestimation of sedentary behavior cannot be excluded, since patients were aware of being monitored and may have been more active and less sedentary than usual. Fourthly, while FMI provides an objective measure of total body fat mass adjusted for height, it does not capture regional fat distribution, specifically visceral fat, which has distinct implications for cardiovascular health [44]. Future studies should examine how sedentary lifestyle contributes to visceral fat accumulation. Finally, like many other studies on sedentary life, our analysis cannot differentiate between strictly sedentary time and light physical activity, such as short bouts of walking or standing, which needs to be considered when interpreting the results. Additionally, sedentary time was analyzed as a global measure, but different types of sedentary behavior (e.g., occupational vs. leisure screen time) may have distinct physiological or behavioral implications, and the lack of this contextual information should be considered when interpreting our findings.

## Conclusions

Our results reveal a high prevalence of unfavorable body composition (FMI) in CAD patients, persisting even after completion of phase II CR. This is associated with other risk factors, including hypertension and impaired lipid levels, which may increase the risk of recurrent cardiovascular events. Moreover, sedentary behavior, irrespective of age, sex, and exercise capacity, is associated with higher FMI. These findings underscore the need for effective intervention strategies specifically targeting sedentary behavior, which could help improve body composition and reduce obesity-related risk in patients with established CAD.

## Data Availability

The raw data supporting the conclusions of the manuscript will be made available by the authors upon reasonable request.

## Abbreviations

ACEi/ARB: Angiotensin-converting enzyme inhibitors and angiotensin receptor blockers
BMI: Body mass index
CAD: Coronary artery disease
CV: Cardiovascular
CVD: Cardiovascular disease
CPET: Cardiopulmonary exercise testing
CR: Cardiac rehabilitation
DXA: Dual-energy radiographic absorptiometry
FMI: Fat mass index
SGLT2: Sodium-glucose cotransporter 2
LIPA: Light intensity physical activity
MRA: Mineralcorticoid receptor antagonists
MVPA: Moderate to vigorous physical activity
RER: Respiratory exchange ration
SMART: Second manifestation of arterial disease
VE/VCO2: Minute ventilation/carbon dioxide production slope
VO_2 _ peak: Peak oxygen consumption
